# Diminished Self-Chaperoning Activity of the ΔF508 Mutant of CFTR Results in Protein Misfolding

**DOI:** 10.1371/journal.pcbi.1000008

**Published:** 2008-02-29

**Authors:** Adrian W. R. Serohijos, Tamás Hegedűs, John R. Riordan, Nikolay V. Dokholyan

**Affiliations:** 1 Department of Physics and Astronomy, University of North Carolina Chapel Hill, Chapel Hill, North Carolina, United States of America; 2 Department of Biochemistry and Biophysics, University of North Carolina Chapel Hill, Chapel Hill, North Carolina, United States of America; 3 Cystic Fibrosis Research Center, University of North Carolina Chapel Hill, Chapel Hill, North Carolina, United States of America; Harvard University, United States of America

## Abstract

The absence of a functional ATP Binding Cassette (ABC) protein called the Cystic Fibrosis Transmembrane Conductance Regulator (CFTR) from apical membranes of epithelial cells is responsible for cystic fibrosis (CF). Over 90% of CF patients carry at least one mutant allele with deletion of phenylalanine at position 508 located in the N-terminal nucleotide binding domain (NBD1). Biochemical and cell biological studies show that the ΔF508 mutant exhibits inefficient biosynthetic maturation and susceptibility to degradation probably due to misfolding of NBD1 and the resultant misassembly of other domains. However, little is known about the direct effect of the Phe508 deletion on the NBD1 folding, which is essential for rational design strategies of cystic fibrosis treatment. Here we show that the deletion of Phe508 alters the folding dynamics and kinetics of NBD1, thus possibly affecting the assembly of the complete CFTR. Using molecular dynamics simulations, we find that meta-stable intermediate states appearing on wild type and mutant folding pathways are populated differently and that their kinetic accessibilities are distinct. The structural basis of the increased misfolding propensity of the ΔF508 NBD1 mutant is the perturbation of interactions in residue pairs Q493/P574 and F575/F578 found in loop S7-H6. As a proof-of-principle that the S7-H6 loop conformation can modulate the folding kinetics of NBD1, we virtually design rescue mutations in the identified critical interactions to force the S7-H6 loop into the wild type conformation. Two redesigned NBD1-ΔF508 variants exhibited significantly higher folding probabilities than the original NBD1-ΔF508, thereby partially rescuing folding ability of the NBD1-ΔF508 mutant. We propose that these observed defects in folding kinetics of mutant NBD1 may also be modulated by structures separate from the 508 site. The identified structural determinants of increased misfolding propensity of NBD1-ΔF508 are essential information in correcting this pathogenic mutant.

## Author Summary

Deletion of a single residue, phenylalanine at position 508, in the first nucleotide binding domain (NBD1) of the Cystic Fibrosis Transmembrane Conductance Regulator (CFTR) is present in approximately 90% of cystic fibrosis (CF) patients. Experiments show that this mutant protein exhibits inefficient biosynthetic maturation and susceptibility to degradation probably due to misfolding of NBD1 and the resultant incorrect interactions of other domains. However, little is known about the direct effect of the Phe508 deletion on NBD1 folding. Here, using molecular dynamics simulations of NBD1-WT, NBD1-F508A, and NBD1-ΔF508, we show that the deletion of Phe508 indeed alters the kinetics of NBD1 folding. We also find that the intermediate states appearing on wild type and mutant folding pathways are populated differently and that their kinetic accessibilities are distinct. Moreover, we identified critical interactions not necessarily localized near position 508, such as Q493/P574 and F575/F587, to be significant structural elements influencing the kinetic difference between wild type and mutant NBD1. We propose that these observed alterations in folding kinetics of mutant NBD1 result in misassembly of the whole multi-domain protein, thereby causing its premature degradation.

## Introduction

**Figure 1:**
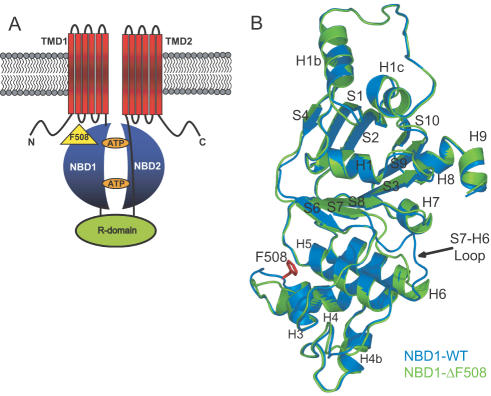
CFTR. (A) CFTR protein consists of nucleotide-binding domains (NBD1 and NBD2), transmembrane domains (TMD1 and TMD2), and a regulatory domain (R domain). Deletion of F508 in NBD1 occurs in ∼90% of CF patients. (B) Superimposed NBD1-WT (PDB ID: 2BBO [12]; blue cartoon) and NBD1-ΔF508 (PDB ID: 1XMJ [10]; green cartoon) structures. Secondary structures of the two proteins overlap. Most notable difference between the mutant and wildtype structures is the S7-H6 Loop, a region separate from the deleted F508.

CF is the most common autosomal inherited disease with high morbidity among Caucasians. CF patients have altered epithelial ion transport that leads to decreased hydration of epithelial surfaces in the gut, kidney, pancreas, and airways [1]. Decreased surface liquid volume impairs mucociliary clearance which in turn leads to respiratory bacterial infection [2],[3]. Chronic pulmonary damage caused by bacterial infection dramatically decreases patients' life expectancies. The absence of a functional ABC protein, CFTR, from apical membranes of epithelial cells is the basis of this pathophysiology in cystic fibrosis [4],[5]. CFTR is a multidomain, integral membrane protein containing two transmembrane domains, two nucleotide-binding domains (NBD1 and NBD2), and a regulatory region (R domain) (Figure 1).

Although more than 1,400 mutations are known in CFTR (http://www.genet.sickkids.on.ca/cftr), approximately 90% of CF patients carry the allele with deletion of the codon for phenylalanine at position 508 [6], which is located in the first nucleotide-binding domain (NBD1) (Figure 1). Experimental studies suggest that the CFTR^ΔF508^ may be arrested at two stages during its biogenesis. First, the loss of the Phe508 backbone may shift a fraction of the NBD1s of nascent CFTR^ΔF508^ off the wild type folding pathway, causing misfolding and eventual rapid degradation [7]–[9]. Interestingly, recent studies show no significant structural difference between the wild type and mutant NBD1 structures nor in their thermodynamic stabilities [10]. Second, the absence of the Phe508 side-chain prevents the correct post-translational assembly of all CFTR domains [11]. The detailed structural origin of the perturbed kinetics of NBD1 leading to its co-translational arrest is unknown.

Nucleotide-binding domains of ABC proteins are highly conserved in sequence and structure. NBDs contain a typical F1 ATPase core subdomain, which consists of an α-helix surrounded by antiparallel β-sheets [9],[12]. This region contains the conserved Walker A and B motifs that are involved in binding ATP. The α-helical subdomain contains the ABC-signature motif important for ATP hydrolysis (Figure 1). From X-ray structures of bacterial transporters, the α-helical subdomain is also known to mediate contact with the transmembrane domains [13],[14].

Folding of multidomain proteins is aided by molecular chaperones to prevent and correct improper (non-native) associations between solvent-exposed hydrophobic regions. Smaller single-domain proteins correct and prevent formation of improper contacts through a sequence of partial folding-unfolding events en route to the native state. This sequence of partial folding-unfolding events reflects the ability of single-domain proteins to self-chaperone their folding. In NBD1, the attenuated refolding of the recombinant ΔF508 mutant is consistent with the notion that Phe508 reduces the activation energy of NBD1 folding *in vivo* as well as *in vitro* [11]. Lowering of the activation energy increases the folding rate, which in turn reduces the folding time for NBD1. Reduction of the folding lessens the propensity of NBD1 to correct the malformed contacts in the intermediate states. Here we propose that Phe508 deletion decreases NBD1's self-chaperoning capability.

To investigate the effect of the Phe508 deletion on the stability, dynamics and kinetics of NBD1, we performed equilibrium dynamics simulations and folding simulations of NBD1-WT and NBD1-ΔF508. Our analysis shows that there is no significant difference in their stability and equilibrium dynamics, which agrees with experiments. However, even in the presence of correcting mutants (G550E, R553Q, and R555K) [10], [15]–[17] in our model of NBD1-ΔF508, we still observe a significant change in dynamics at the folding transition. We further explore the difference in the folding transition by performing 300 folding simulations each for NBD1-WT and NBD1-ΔF508. We also perform simulations of another mutant NBD1-F508A to serve as control. These simulations allow the comparison of the mutant and wild type folding probabilities, their intermediate states, the structures of these intermediate states, and their folding pathways. Finally, we identify contacts between residues in NBD1 critical to its folding dynamics that are perturbed by Phe508 deletion, thus increasing the propensity of NBD1-ΔF508 to misfold.

## Results

### Thermodynamics of NBD1

**Figure 2:**
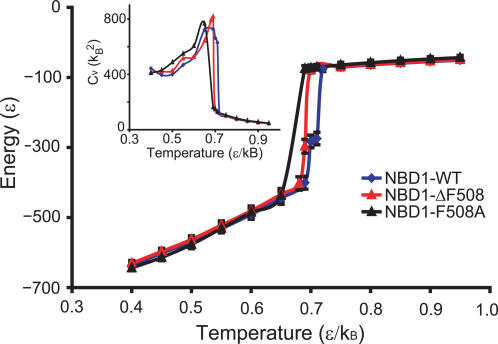
Thermodynamics of NBD1-WT, NBD1-F508A, and NBD1-ΔF508. Energy is calculated from long equilibrium simulations (10^6^ time units) of NBD1-WT, NBD1-F508A, and NBD1-ΔF508 crystal structures. Error bars represent±standard deviation. (Inset) The specific heat is calculated as *C_v_* = (〈*E* ^2^〉−〈*E*〉^2^)/*T* ^2^. Wild type and mutant NBD1s exhibit similar thermodynamic states but different dynamics near the folding transition.

To determine the equilibrium dynamics and stabilities of the wild type and mutant NBD1, we perform equilibrium simulations (10^6^ time units∼0.5 millisecond [18]) of wild type and mutant NBD1 using discrete molecular dynamics [19],[20] (see Methods). From the equilibrium simulations, we calculate the thermal denaturation curve of both NBD1-WT and NBD1-ΔF508 (Figure 2) and observe two stable thermodynamic states, folded and unfolded. In agreement with previous experimental studies by denaturation experiments [7]–[9], the stabilities of wild type and ΔF508 NBD1 are not significantly different.

The slope at the transition temperature of the wild type (*T_m_*∼0.68 ε/*k_B_*) is 9838 *k_b_* and the slope at the transition temperature of the mutant (*T_m_*∼0.70 ε/*k_B_*) is 16201 *k_b_* (ε∼1–2 kcal/mol and *k_B_* is the Boltzman factor; see Methods for further discussion on units). This shift in slope at the transition temperature indicates a difference in folding cooperativity of NBD1-WT and NBD1-ΔF508 and therefore a difference in folding kinetics.

### Folding Kinetics of NBD1

Folding is a stochastic process, thus to investigate in detail the difference in folding kinetics and dynamics of NBD1-WT and NBD1-ΔF508, we perform 300 folding simulations on each of the structures. Starting from fully unfolded chains of NBD1-WTand NBD1-ΔF508, we progressively reduce the temperature of the system to simulate thermal folding (see Methods). We find the folding probability [21] (number of runs that lead to the native structure/number of total folding simulations) of wild type to be 33±3% while that of the mutant is 13±2% (see Methods). The ratio of NBD1-WT and NBD1-ΔF508 correlates with the ratio of their folding yields derived from folding experiments. Folding yields of NBD1-WT is approximately twice that of NBD1-ΔF508 in the temperature range 10°C to 22°C [9]. Folding simulations of our control structure NBD1-F508A yield a folding probability of 26±4% which is intermediate to that NBD1-WTand NBD1-ΔF508. This folding probability value is in agreement with experimental studies showing intermediate folding efficiencies and maturation levels of NBD1-F508A relative to NBD1-WT [9],[11].

**Figure 3:**
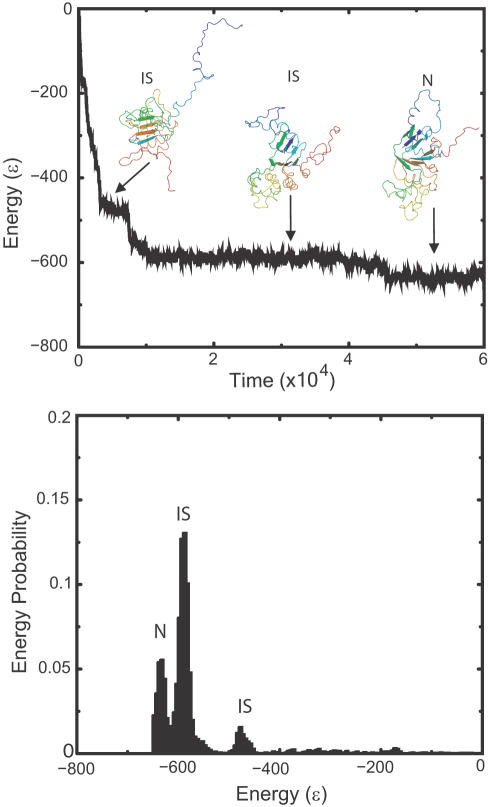
NBD1 folding. We perform annealing simulations in which the temperature is decreased to facilitate folding of NBD1. Shown is a time evolution of energy starting from unfolded to native (N) states. As the protein proceeds towards it native state, it goes through metastable folding intermediate states (IS), which are observed as peaks in the energy probability distribution.

To investigate the molecular origin of the difference in folding yields and probabilities, we map the folding pathways of NBD1-WT, NBD1-F508A, and NBD1-ΔF508 by identifying their metastable folding intermediate states. The folding intermediate states of a folding trajectory are exhibited as peaks in the energy probability distributions (Figure 3; Figure S1). Thus, dominant intermediate states in the folding pathways are peaks in the average energy probability distributions (see Methods; Figure 3). The average energy probability distributions of wild type and the mutant are significantly different (Kolmogorov-Smirnov test; P-value<1.4×10^−292^), which suggests a significant difference in the folding kinetics of wild type and mutant NBD1. The dominant intermediate states are listed in Table S1. The average fraction of native contacts of NBD1 structures in an intermediate state follows a distinct distribution (Figure S2), thus, an intermediate state identified using energy as the folding reaction coordinate, forms a distinct collection of NBD1 conformations.

We find that some intermediate states are accessible only by either NBD1-WT (S6 and S9) or NBD1-ΔF508 (S5 and S10), further suggesting that Phe508 deletion leads the mutant to off-folding pathways (see below). While states S2, S3, S4, S7, and S8 are both traversed by NBD1-ΔF508 and NBD1-WT, their time occupancies (length of time NBD1 spends in an intermediate state) are different (Figure 3B). Since time occupancies are proportional to the free energy barriers between intermediate states, these observations suggest that the Phe508 deletion significantly perturbs the NBD1 folding free energy landscape.

### Folding Pathways

**Figure 4:**
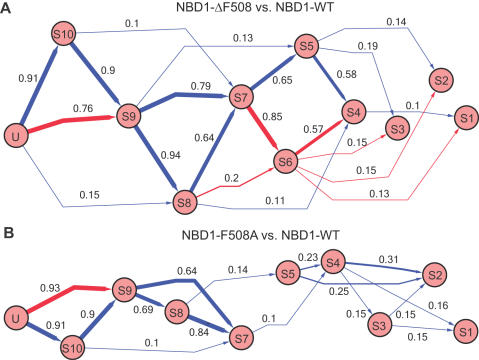
Comparison of the folding pathways of wild type NBD1 and its mutants. Transition probabilities between intermediate states are estimated by counting the number of trajectories that exhibit the transition (Figure S2). Shown are the difference transition probabilities between (A) NBD1-ΔF508 and NBD1-WT and between (B) NBD1-ΔF508 and NBD1-WT. Blue edges denote transitions dominant in the mutant folding pathway, while red edges denote transitions in the wild type folding pathway.

To determine the difference between the sequence of folding events of the wild type, ΔF508, and the F508A control, we estimate the probability of transitions between intermediate states (see Methods and Figure S3). The difference in transition probabilities of NBD1-WT, NBD1-ΔF508, and NBD1-F508A is shown in Figure 4. The transition probabilities show some states accessible only to either wild type or mutant NBD1. The difference in state accessibilities between the two indicates a difference in contact pattern formation (nucleation events), which could cause the observed difference in folding yields.

We calculate the most dominant folding pathways in wild type and mutant NBD1. The most dominant path in wild type follows a sequence of transition Unfolded→S10→S8→S7→S5→S4→S1, while the dominant path in the mutant follows the sequence of transitions Unfolded→S9→S8→S7→S6→S4→S1. Thus, NBD1-WTand NBD1-ΔF508 undergo different sequences of folding events.

### Structures of Intermediate States

**Figure 5:**
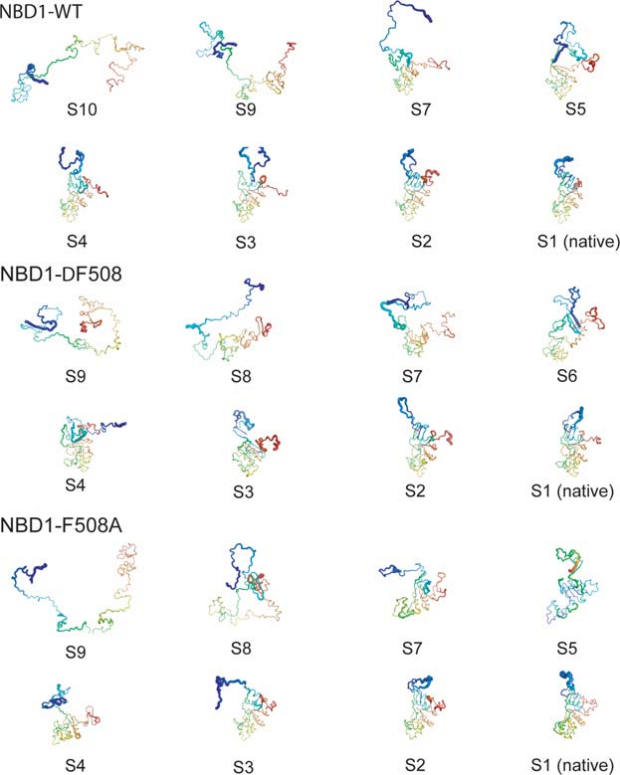
Structures of folding intermediates. To identify the structure most representative of an intermediate state, we cluster the structures within the folding intermediate. Shown are the centroids of the dominant clusters. Diameter of the backbone cartoons is proportional to the average per residue root-mean-square deviation (RMSD) of the structures within the intermediate state. Blue and red represent N- and C-termini, respectively.

**Figure 6:**
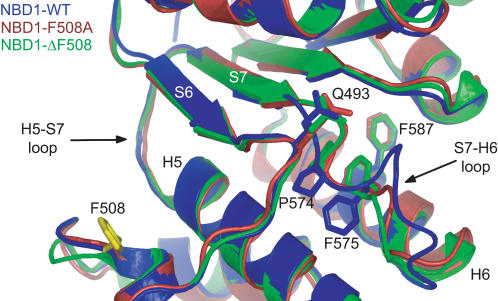
Contacts in NBD1-WT that perturbed in the F508A and ΔF508 mutants. Difference between average contact frequencies of structures within intermediate states shows malformed contacts in NBD1-ΔF508 (green) compared to NBD1-WT (blue). These identified malformed contacts in the mutants are critical determinants of NBD1 folding kinetics. In particular, P574 interacts with Q493 in wild type but not in the mutant. Also, F575 interacts with F587 in mutant but not in wild type. Redesigning these contacts to their wild type interactions in the ΔF508 background can potentially rescue NBD1-ΔF508.

Because of the reduction in dimensionality of the folding process when energy is used as a reaction coordinate, each intermediate state represents an ensemble of NBD1 structures. To identify the primary structural characteristics of each intermediate state, we clustered structures in the corresponding state and calculated the frequency of contacts formed between pairs of residues (Figure 5; see Methods). In all intermediate states, we find the most notable structural difference between NBD1-WT and NBD1-ΔF508 occurs in the S7-H6 loop. For example, P574 interacts with Q493 in wild type but not in the mutant. Also, F575 interacts with F587 in the mutant but not in wild type (Figure 6). This pattern of contact formation reflects the difference in NBD1-WT and NBD1-ΔF508 crystal structures that is embedded in the interactions defined according to structure. Additionally, residue pairs that have similar interactions (i.e., attractive or repulsive) in the wild type and mutant crystal structures still exhibit different contacts in the folding intermediate states. These results show that the pattern of transient contact formation in the wild type is also perturbed by Phe508 deletion. This class of residue pairs include Q525/E585 and C524/I586.

### Frustrated Folding Trajectories

We observe a number of folding trajectories reaching native energies (∼630 ε) and within a 2.5 Å root-mean-square deviation (RMSD) with respect to the native structure, but the resulting topological wiring of the secondary structures is incorrect. The “miswiring” consistently occurs in the H5-S6 loop. Interestingly, this H5-S6 loop is in the immediate neighbourhood of the loop containing Phe508. This suggests “weak” regions in NBD1 that are intrinsically prone to misfolding.

### Computational Rescue of ΔF508 Mutant

To verify that the identified contact pairs (Q493/P574 and F575/F587) found in the S7-H6 loop are indeed critical in the kinetics of NBD1, we revert their interactions in NBD1-ΔF508 to their interactions in NBD1-WT and perform folding simulations. In the case of the Q493/P574 pair, the residues are in close proximity in NBD1-WT but not in NBD1^ΔF508^, thus we changed the interaction between Q493 and P574 in NBD1^ΔF508^ from repulsive to attractive to mimic a possible rescuing mutation. Folding simulations of “rescued” NBD1-ΔF508 yield a folding probability of 19±2%. On the other hand, residues F575 and F508 are in close contact in NBD1-ΔF508 but not in NBD1-WT, thus we reverted their interaction in NBD1-ΔF508 from attractive to repulsive. Folding simulations of the second “rescued” NBD1-ΔF508 yield a folding probability of 20±2%. These folding probabilities of the two “rescued” NBD1-ΔF508s are higher than the 13±2% folding probability of the original NBD1-ΔF508, which supports our findings that the contacts between Q493 and P574 and between F575 and F587 are indeed critical to NBD1 folding.

## Discussion

Deletion of Phe508 in CFTR NBD1 is the most frequent mutation in patients with cystic fibrosis. Proteins with ΔF508 mutation in the first nucleotide binding domain NBD1 can not mature resulting in absence of functional CFTR from the plasma membrane. The molecular mechanism leading to this pathological situation is unknown. No significant difference in thermodynamic stabilities was experimentally observed between wild type and mutant NBD1 [7]–[10]. Crystal structures of NBD1-WT, NBD1-F508A, and NBD1^ΔF508^ are also practically identical except for the S7-H6 loop (Figure 1B) [9],[10],[12]. However, the folding yields of the wild type and mutant were observed to be different [9],[10],[12] suggesting that Phe508 deletion alters the folding kinetics and dynamics of NBD1. Using computational tools, we find that indeed Phe508 deletion causes the mutant to follow a different folding pathway from that of the wild type. We also investigate the molecular origin of NBD1^ΔF508^ aberrant folding.

Our model agrees with the experimental studies where wild type and mutant NBD1 did not exhibit significant thermodynamic difference. Thermal denaturation curves of both wild type and mutant were calculated from long equilibrium dynamics simulations (∼0.5 millisecond) (Figure 2). We find that there is no significant difference in the stabilities of wild type and mutant NBD1, which agrees with experimental observations showing unaltered thermodynamic stability upon Phe508 deletion from NBD1 [9],[10],[12]. The thermal denaturation curves likewise show a slight shift in the melting temperature, which agrees with the minor change in melting temperature from 49°C to 46°C upon deletion of Phe508 [8]. The agreement between our computational results and experimental studies validates our model and methodology. The shift in *T_m_* reflects the attenuated folding of NBD1, consistent with the notion that Phe508 reduces the activation energy of NBD1 folding *in vitro* and *in vivo* [11]. These observations do not contradict biochemical and functional measurements that show rescued complete ΔF508 CFTR has a temperature-sensitive stability defect in post-ER compartments [22]. While the stability of NBD1 may be minimally perturbed upon Phe508 deletion, the impaired interaction of NBD1 with the rest of CFTR could still destabilize the whole CFTR. Misfolding of NBD1 may prevent its proper interaction with other CFTR domains.

Interestingly, even a simplified protein model (4-bead representation for non-aromatic residues and 5-bead for aromatic residues) and a simplified potential (Go̅-type interaction) show a significant difference in NBD1-WT and NBD1^ΔF08^ kinetics. Indeed the multiple folding simulations of NBD1 models show significantly higher folding efficiency for the wild type than the ΔF508 mutant, which correlate with some experimental studies that found higher folding yield for wild type NBD1 than its ΔF508 mutant [8]. A relevant control of our simulation protocol and modeling assumptions is the folding simulation of the NBD1-F508A that yields a folding probability of 26±4%, which is higher than that of NBD1^ΔF08^ but lower than that of NBD1-WT. This observation again correlates with the measured folding efficiencies of isolated NBD1 and maturation levels of whole CFTR [9],[11].

We also find that the folding time of NBD1^ΔF508^ mutant is smaller than that of wild type, suggesting an increase in the effective folding rate upon Phe508 deletion. Consistent with the notion that the refolding of recombinant ΔF508 mutant reduces the activation energy of NBD1 folding *in vivo* as well as *in vitro* [11]. The reduction in folding time diminishes the propensity of NBD1 to correct and prevent malformed contacts in the intermediate states. Thus, mutant NBD1 has a diminished self-chaperoning activity.

What is the origin of this loss in self-chaperoning capacity by the Phe508 mutant? To answer this question, we identify and compare the folding intermediates accessed by wild type and mutant *en route* to the native state. Calculation of transition frequencies from one state to another reveal differences in the accessibilities of intermediate states (Figure 5). Some intermediate states are only accessible in either wild type or mutant. These observations suggest that the progression of contact formation is different in the two structures. We also determine the primary differences in the progression of contact formation by calculating the most frequent contacts formed within the intermediate states. Between wild type and mutant NBD1 intermediates, the most notable differences are in the interaction between amino acid pairs F575/F587 and Q493/P574. Contact between Q493 and P574 is consistently formed in the intermediate states of the wild type but not of the mutant (Figure 4). Interestingly, the P574S mutation has been observed in a CF family also possessing the ΔF508 mutation but without significant pulmonary or pancreatic disease. The solubilizing F494N mutation, which is adjacent to Q493, has also been shown to partially correct the folding defect of CFTR-ΔF508 [23]. The mutations P574S and F594N may promote contact formation between Q493 and P574 during NBD1^ΔF508^ folding, thus rescuing NBD1^ΔF508^ from the misfolding defect. Interestingly, Thibodeau *et al.* found that NBD1^F508W^, the only F508X mutant with a lower folding efficiency than NBD1^ΔF508^, can be rescued by introducing the compensating mutation W496F, which is exactly in the same loop that contains Q493 and F594 [9].

A drawback that may arise from using Go̅ is that the properties of a protein are determined solely by its geometry, an assumption that apparently deviates from the observation that sequence is also a key determinant of folding properties. However, this potential drawback is not limiting in our study of the folding kinetics of wild type NBD1 and its mutants. The nuanced effect of a mutation or deletion at position 508 is already reflected in the S7-H6 loop conformation of NBD1-WT, NBD1-F508A, and NBD1^ΔF508^ crystal structures.

Changes in NBD1 folding kinetics have been shown earlier experimentally. Qu *et al.* observed dramatic changes in the temperature sensitivity of the folding process in the absence of Phe508 [8]. In the temperature and protein concentration range used in the refolding experiments, mutant NBD1 reached the native state less efficiently compared to wild type. The mutant NBD1 aggregated faster and to a larger extent, as observed by light scattering of the samples. In contrast, Lewis *et al.* observed no difference in folding of wild type and ΔF508 NBD1 monitored by CD spectroscopy or intrinsic Trp fluorescence [10]. This apparent disparity may be due to a substantial difference in experimental conditions of these two refolding experiments. While the former laboratory performed these experiments at different temperatures incubating the samples overnight, the latter made measurements immediately after dilution of the denaturant over a timescale of minutes. In the experiments of Qu *et al.* [7],[8], there is a possibility that the two constructs have different solubilities. On the other hand, Lewis and coworkers' studies of folding kinetics were carried out only at room temperature, where the differences between the refolding of the two different domains are already attenuated compared to lower temperature (10°C–16°C) [10].

### Conclusion

Our results reveal the intrinsic property of NBD1^ΔF508^ to fold improperly and raise the possibility of redesigning NBD1^ΔF508^ to rescue it from misfolding. In case of the contact that is found in wild type but not in the ΔF508 mutant (e.g., Q493/P574), one can find amino acid substitutions that promote interaction between this pair of residues (Q493/P574). On the other hand, for the contact found only in the ΔF508 mutant but not in wild type (e.g., F475/F587), candidate rescue mutants are those that destabilize the interaction between this residue pair (F475/F587). Knowing the molecular details of the altered folding in the case of the mutant domain also provides a basis for design of small molecules to correct the most prevalent and pathogenic mutation in CFTR.

## Methods

### Protein Model

To access time scales of NBD1 folding, we use a simplified protein model but still maintain important features of the protein such as side-chain packing. Amino acid residues were modelled as follows: (1) glycines are represented by three beads (-N, C_α_, C′); (2) phenylalanine, tyrosine, tryptophan, and histidine by five beads (-N, C_α_, C′, C_β_, C_γ_), and (3) all other residues by four beads (-N, C_α_, C′, C_β_) [24]. This protein model successfully described protein aggregation [24]. In the simulations, we use PDB ID: 2BBO, PDB ID: 1XMI and PDB ID: 1XMJ [10] as models of NBD1-WT, NBD1-F08A, and NBD1-ΔF508, respectively. The missing loop between E403 and L436 in both wild type and mutant NBD1 is reconstructed using a loop-search algorithm in SYBYL (Tripos Assoc. Inc, St. Louis, MO).

### Thermodynamics of the NBD1 Domain

Using discrete molecular dynamics [19],[20], long equilibrium simulations at various temperatures were performed to investigate the equilibrium dynamics of the CFTR NBD1. Interactions between beads were defined using the Go̅-model [25]. In the Go̅-model, interactions between residues are determined from the native structure of known NBD1 crystal structures. Pairwise, square-well interactions were assigned between beads in the model according to contacts formed in the native state. Specifically, two residues are said to be in contact if their atoms (excluding hydrogen) are within a distance of 4.5 Å. The strength of the interaction between residues in contact (denoted hereon as ε) defines the energy units. Physically ε∼1–2 kcal/mol, which is approximately a contribution to protein stability from a hydrogen bond. The temperature is measured in units of ε/*k_b_*, where *k_b_* is the Boltzmann constant. The time unit (tu) is estimated to be the shortest time between particle collisions in the system (∼0.1 nanosecond). From long equilibrium simulations of 10^6^ tu, we were able to access the long time-scale dynamics of the CFTR NBD1 in the order of 0.5 millisecond. Each equilibrium simulation consumed approximately 300 CPU hours.

### Folding Simulations

We perform 300 folding simulations for each NBD1-WT, NBD1-F508A, and NBD1-ΔF508. Starting from fully unfolded chains, the temperature of the system is progressively reduced to allow NBD1 to fold to its native structure. Folding simulations proceeded until τ*_max_*∼60,000 tu (time units), which is chosen to be longer than the typical folding time of the studied sequences [21]. A similar criterion was employed in the studies calculating the folding probability of proteins [26]. The NBD1 structure in a folding run is considered folded when (1) its energy is less than or equal to −620ε (the energy of the native state), (2) its structure is within 2.5 Å RMSD from the native, and (3) the structure possesses correct topological wiring of the secondary structure elements. To estimate the error in folding probabilities, each folding trajectory is considered a Bernoulli trial with a binary outcome, folded or unfolded. The variance of a Bernoulli process is σ^2^ = *p*(1−*p*)/*n*, where *p* is probability and *n* is the total number of trials.

### Identification of Metastable Intermediate States

To identify the positions of intermediate states, a sum of multiple Gaussian curves 
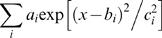
 is fitted to the average energy probability distribution of successfully folded runs. *a_i_*, *b_i_*, and *c_i_* are the center, standard deviation and height of the *i*th Gaussian curve, respectively.

### Kinetic Accessibilities of Intermediate States and Most Likely Paths

We estimate probability of transitions between states by counting the trajectories that underwent such transition. The sum of probabilities of paths emanating from a given state is normalized to 1, which physically means that the system always exits from its current intermediate state. The transition probabilities represent independent conditional probabilities, thus the most likely path from the unfolded state to the native is estimated by multiplying the probabilities of the traced edges.

### Structural Characterization of Intermediate States

We calculated a contact matrix for each structure in the intermediate state. An element of the contact matrix is 1 when two residues were within 4.5 Å or 0 otherwise. Dominant contacts between pairs of residues in NBD1 are determined from the average contact matrix of all the structures.

## Supporting Information

Figure S1 **Energy probability distributions averaged over all successful folding trajectories.**
Positions of metastable intermediate states are identified by fitting a sum of gaussian distributions. Each gaussian curve corresponds to a folding intermediate state.
(0.57 MB TIF)

Figure S2 **Distribution of fraction of native contacts.**
For a given state, we calculated the average fraction of native contacts (Q) coming from a particular folding trajectory. The normalized distribution of Q shows that the states defined using energy are structurally distinct.
(0.65 MB TIF)

Figure S3 **NBD1 folding pathways.**
Probability of kinetic transitions between intermediate states of NBD1-WT, NBD1-ΔF508, and NBD1-F508A. The probability of exiting a state is normalized to 1. The thickness and warmth of the transition edges are rendered proportional to the probability value.
(0.99 MB TIF)

Table S1 **Intermediate states of NBD1-WT defined from the peaks of the energy probability distribution.**
Also shown are the average root-mean-square deviations (RMSD) of intermediate state structures with respect to the native structure.
(0.04 MB DOC)
