# Supplementary figures and images for: Diminished Self-Chaperoning Activity of the ΔF508 Mutant of CFTR Results in Protein Misfolding
Source: PLoS Comput Biol. 2008 Feb 29;4(2):e1000008. doi: 10.1371/journal.pcbi.1000008 (PMC2265529; doi:10.1371/journal.pcbi.1000008)

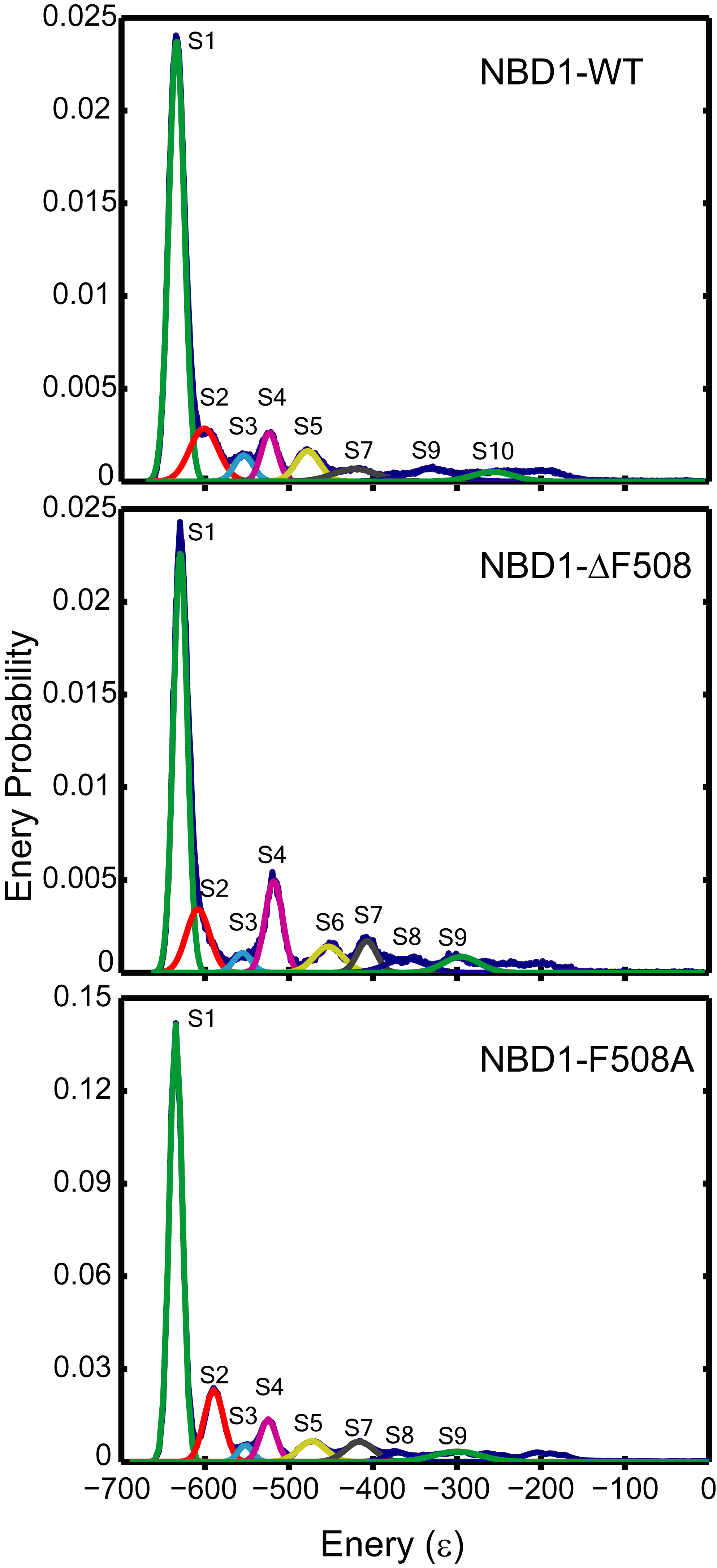

Supplement: Figure S1 — Energy probability distributions averaged over all successful folding trajectories. Positions of metastable intermediate states are identified by fitting a sum of gaussian distributions. Each gaussian curve corresponds to a folding intermediate state. (0.57 MB TIF) [file pcbi.1000008.s001.tif]

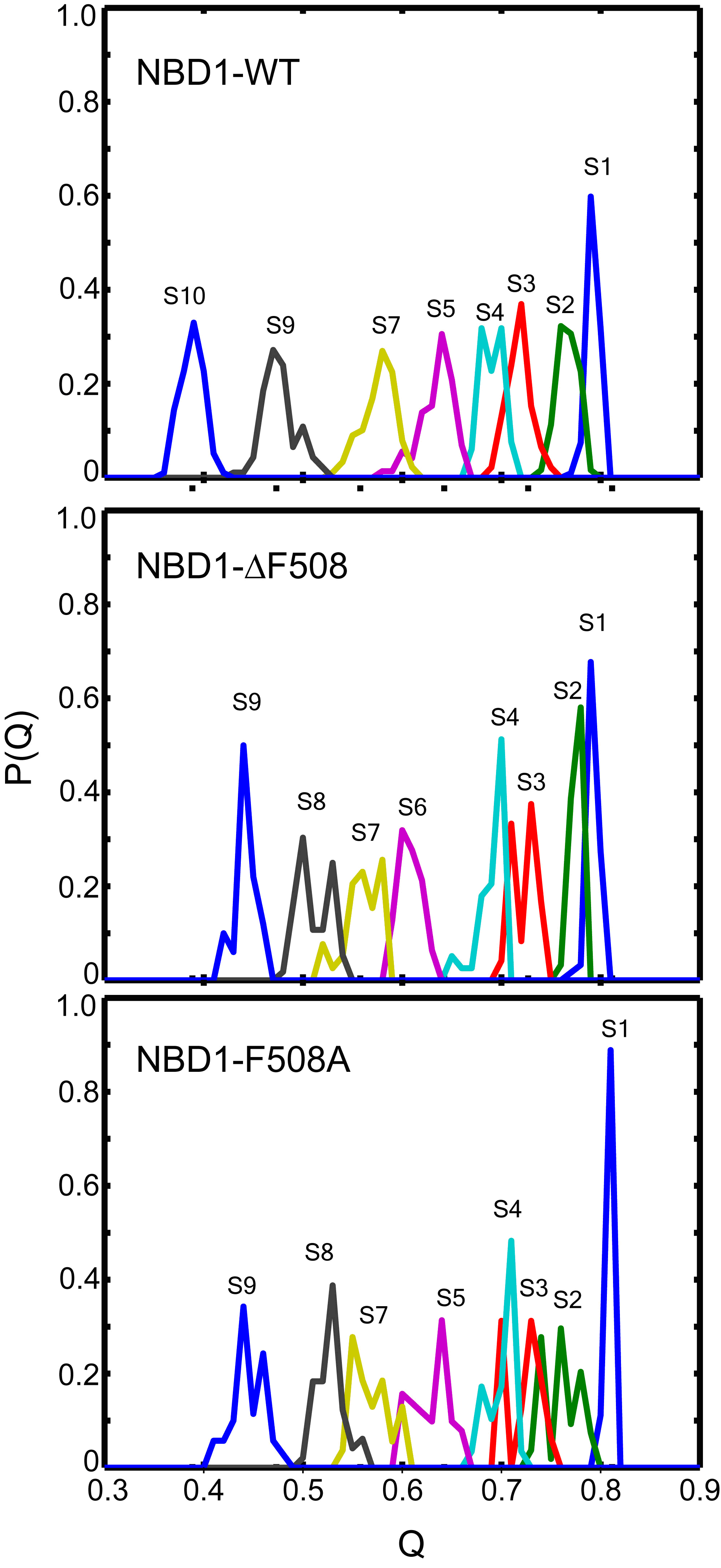

Supplement: Figure S2 — Distribution of fraction of native contacts. For a given state, we calculated the average fraction of native contacts (Q) coming from a particular folding trajectory. The normalized distribution of Q shows that the states defined using energy are structurally distinct. (0.65 MB TIF) [file pcbi.1000008.s002.tif]

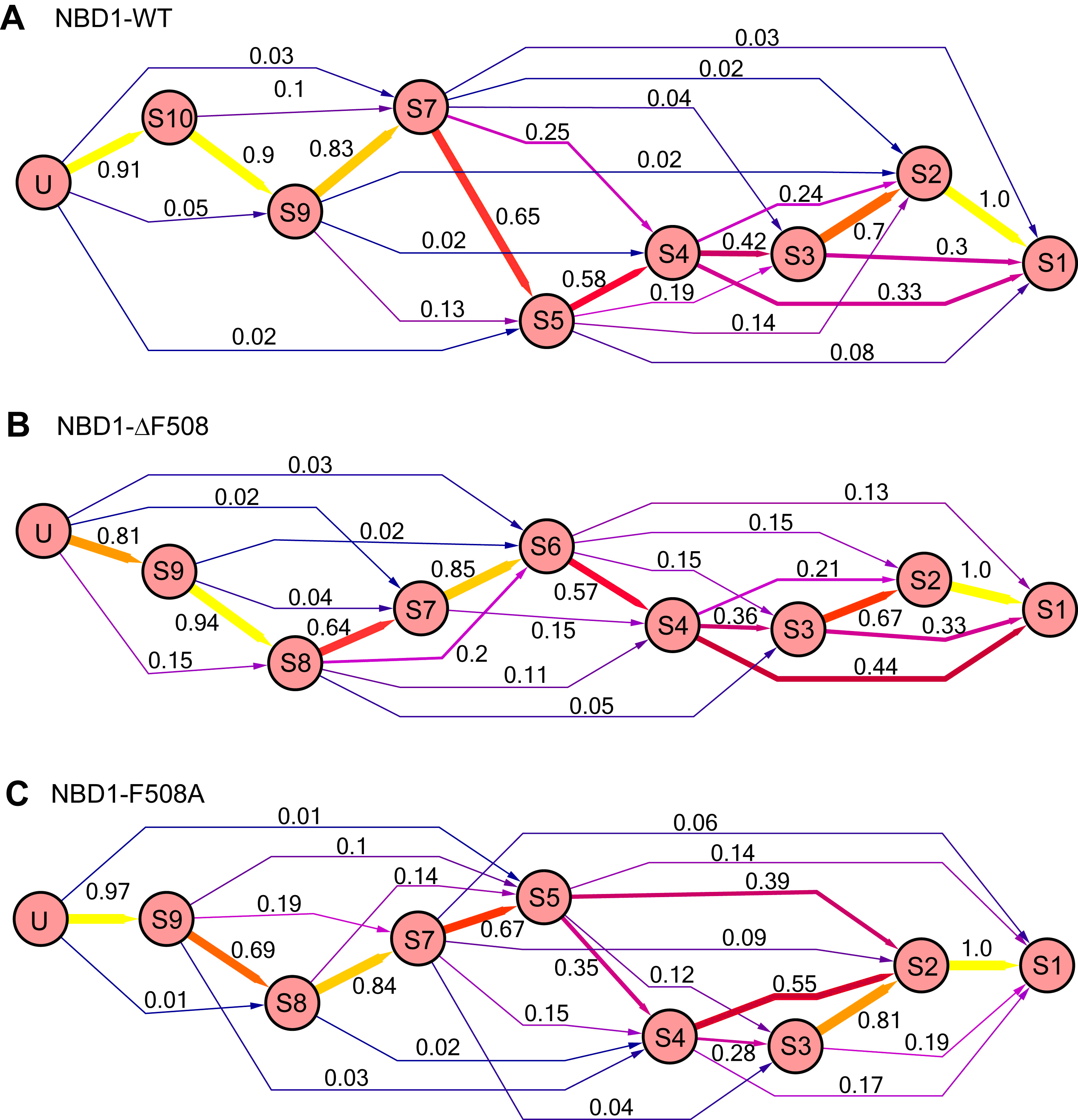

Supplement: Figure S3 — NBD1 folding pathways. Probability of kinetic transitions between intermediate states of NBD1-WT, NBD1-ΔF508, and NBD1-F508A. The probability of exiting a state is normalized to 1. The thickness and warmth of the transition edges are rendered proportional to the probability value. (0.99 MB TIF) [file pcbi.1000008.s003.tif]
