# Supplementary material for: Diminished Self-Chaperoning Activity of the ΔF508 Mutant of CFTR Results in Protein Misfolding
Source: PLoS Comput Biol. 2008 Feb 29;4(2):e1000008. doi: 10.1371/journal.pcbi.1000008 (PMC2265529; doi:10.1371/journal.pcbi.1000008)
Supplement: Table S1 — Intermediate states of NBD1-WT defined from the peaks of the energy probability distribution. Also shown are the average root-mean-square deviations (RMSD) of intermediate state structures with respect to the native structure. (0.04 MB DOC) [file pcbi.1000008.s004.doc]

| **State** | **Energy (ε)** | | | **Ave. RMSD^1^ (Å)** | | | **Ave. Q^2^** | | |
| --- | --- | --- | --- | --- | --- | --- | --- | --- | --- |
|  | **NBD1-WT** | **NBD1-**  **ΔF508** | **NBD1-**  **F508A** | **NBD1-WT** | **NBD1-**  **ΔF508** | **NBD1-**  **F508A** | **NBD1-WT** | **NBD1-**  **ΔF508** | **NBD1-**  **F508A** |
| **S1** | -634 (7) | -634 (6) | -634 (5) | 2.9 (0.3) | 2.5 (0.3) | 2.9 (0.2) | 0.79 (0.01) | 0.79 (0.01) | 0.81 (0.01) |
| **S2** | -601 (12) | -613 (10) | -589 (8) | 4.0 (0.6) | 3.2 (0.6) | 5.3 (2.2) | 0.77 (0.01) | 0.78 (0.01) | 0.76 (0.02) |
| **S3** | -554 (8) | -560 (8) | -552 (6) | 6.5 (1.8) | 6.0 (1.6) | 5.5 (1.7) | 0.72 (0.01) | 0.72 (0.01) | 0.72 (0.02) |
| **S4** | -523 (7) | -523 (7) | -525 (7) | 9.2 (2.1) | 9.2 (1.8) | 8.9 (2.2) | 0.69 (0.01) | 0.69 (0.01) | 0.70 (0.01) |
| **S5** | -478 (10) | nd^3^ | -472 (11) | 11.4 (1.7) | nd^3^ | 12.4 (2.2) | 0.64 (0.02) | nd^3^ | 0.63 (0.02) |
| **S6** | nd^3^ | -458 (12) | nd^3^ | nd^3^ | 12.5 (2.2) | nd^3^ | nd^3^ | 0.61 (0.01) | nd^3^ |
| **S7** | -421 (18) | 412 (8) | 416 (11) | 14.7 (2.4) | 15.1 (2.3) | 15.2 (2.6) | 0.58 (0.02) | 0.56 (0.17) | 0.57 (0.02) |
| **S8** | nd^3^ | -366 (16) | -370 (10) | nd^3^ | 17.7 (2.4) | 17.3 (2.1) | nd^3^ | 0.51 (0.15) | 0.53 (0.01) |
| **S9** | -332 (20) | -300 (15) | -299 (20) | 19.5 (2.7) | 21.1 (3.2) | 21.7 (3.1) | 0.48 (0.02) | 0.44 (0.01) | 0.45 (0.02) |
| **S10** | -257 (17) | nd^3^ | nd^3^ | 25.5 (4.4) | nd^3^ | nd^3^ | 0.39 (0.01) | nd^3^ | nd^3^ |

^1^RMSD, root-mean-square deviation with respect to the native structure

^2^Fraction of native contacts.

^3^Intermediate state **n**ot **d**etected.
